# Is There an Adequate Therapeutic Approach to Thyroid Pathology in Patients with Down Syndrome?

**DOI:** 10.3390/diagnostics13233499

**Published:** 2023-11-21

**Authors:** Maria Teresa Murillo-Llorente, Marcelino Pérez-Bermejo, Verónica Llacer-Heredia, Beatriz Tomás-Aguirre, Angel Valls-Arévalo, Francisco Tomás-Aguirre

**Affiliations:** 1 SONEV Research Group, School of Medicine and Health Sciences, Catholic University of Valencia San Vicente Mártir, C/Quevedo nº 2, 46001 Valencia, Spain; mt.murillo@ucv.es; 2 School of Medicine and Health Sciences, Catholic University of Valencia San Vicente Mártir, C/Quevedo nº 2, 46001 Valencia, Spain; veronica.llacer@mail.ucv.es (V.L.-H.); paco.tomas@ucv.es (F.T.-A.); 3 CS Serreria II, Down Syndrome Unit—Clínico-Malvarrosa Department, Social Pediatrics Workgroup SVP, 46022 Valencia, Spain; tomas_bea@gva.es; 4 CS Nazaret Valencia, Clínico-Malvarrosa Department, INCLIVA Nutrition Research Group, 46024 Valencia, Spain; anvallsa@gmail.com

**Keywords:** Down syndrome, thyroid hormones, free thyroxine, thyroid-stimulating hormone

## Abstract

Thyroid dysfunction stands as the most prevalent endocrine disorder in individuals with Down syndrome, particularly showcasing both clinical and subclinical hypothyroidism. TSH and FT4 blood values serve as common diagnostic and treatment adjustment markers. In Down syndrome (DS), hormone values may deviate from those observed in the general population, which may lead to overdiagnosis and consequent iatrogenesis of subclinical hypothyroidism. The objective of this study was to analyze the appropriateness of the replacement therapeutic approach by identifying the TSH and FT4 values that can be considered normal in these patients. Methods: A cross-sectional study was conducted in 503 subjects with DS of both sexes and without age limit drawn from the Health Program for individuals with DS in Valencia (Spain) from February 1993 to November 2021. The exclusion criteria included hyperthyroidism, nodules, tumors, or individuals under treatment with drugs influencing iodine metabolism. The normality of data distribution was assessed using the Shapiro–Wilk test. Outliers were detected using the Reed’s criterion. Hormone values were estimated using quantile regression models for the 2.5th and 97.5th percentiles. Results: The normal values identified were 0.88–11.25 mIU/L for TSH and 0.71–1.63 ng/dL for FT4. The Wald test indicated no significant differences in the reference intervals based on age or sex. Conclusion: The establishment of these values, which, in people with DS, can be considered unique, is of great importance, allowing a watchful waiting attitude to be maintained before starting replacement therapy that is unnecessarily or adjusting medication in diagnosed cases.

## 1. Introduction

The most common chromosomal disorder in our society is Down syndrome (DS) [1,2], so it is crucial to understand the specific characteristics and circumstances that significantly impact health. It affects 700–1000 of live-born infants [2] and presents characteristic clinical signs and symptoms such as intellectual disability, hypotonia [1,2,3], failure to thrive [3,4], macroglossia [3], obesity [1,3,4] and bradycardia [4], among others, which overlap with the clinical features of hypothyroid pathology [3,4].

Thyroid dysfunction is the most frequent endocrine alteration in patients with Down syndrome [1,2,3,4,5], with the presence of subclinical hypothyroidism (SCH or hyperthyrotropinemia), congenital (CH) or acquired (autoimmune or not), and hyperthyroidism [1,5,6] being even more notable. This may be because the glands with anti-NKX2-1 antibodies in Down syndrome are smaller and have fewer follicles and fewer antithyroglobulin-containing colloids compared to the same glands in the general population [1]. Its prevalence exceeds 60% [2,7], depending on age, variations in population sample size, laboratory tests performed to measure the necessary hormones and antibodies, or definitions of thyroid dysfunction used in each study [2,4].

There is great controversy regarding the main thyroid abnormality among patients with Down syndrome. Campos et al. [2] and King et al. [8] consider congenital hypothyroidism as the most prominent [2,8]; however, Whooten et al. [5], Al Aaraj et al. [7], and Aversa et al. [9] state that it is acquired autoimmune hypothyroidisms, such as Hashimoto’s disease (HD), or hyperthyroid disorders, such as Graves’ disease (GD) [5,7,9], that children with this disorder are really susceptible to.

These pathologies have a higher incidence in the population with Down syndrome than in the general population [8], and although the cause of this condition is unknown [10], we must be very clear about when these individuals are genuinely suffering from these disorders and when thyroid hormone values found within the normal range for this syndrome are being considered as abnormal. Several studies, such as those by Amr [1] and Whooten et al. [5], have shown that cases of congenital and subclinical hypothyroidism are being diagnosed and treated in patients with Down syndrome whose only indication of having the pathology is elevated TSH thyroid hormone [1,5] above the values established as normal for the general population.

Several studies classify hypothyroidism, including subclinical (or compensated) hypothyroidism, and hyperthyroidism according to FT4 and TSH values, considering that hypothyroidism exists with an FT4 value < 0.7 ng/dL (low) and a TSH > 5 mIU/L [1,4,7], and subclinical hypothyroidism has an FT4 value between 0.7 and 1.86 ng/dL (normal) and a TSH between >5 mIU/L [1,4,7] and <12 mIU/L [4]. There is controversy in the definition of compensated hypothyroidism since some studies indicate that it would be when TSH is below 20 mIU/L, whereas others differentiate between whether it is between 6 and 10 mIU/L or between 11 and 20 mIU/L [1]. Finally, the most commonly used definition of hyperthyroidism is when there is an FT4 value > 0.7 ng/dL [4,6] and a TSH < 0.5 mIU/L [4].

These values are determined as normal for the general population, but they cannot be applied to individuals with Down syndrome since there is evidence that TSH is usually elevated, but without specific limits, which may encourage overdiagnosis of subclinical hypothyroidism in these patients [1]. We can observe this in studies such as that by Cebeci et al. [10], where the median TSH at the first visit is 10.40 (19.4) mIU/L and FT4 is 1.18 (0.43) ng/dL. On the other hand, Amr [1] noted that several studies showed TSH > 10 mIU/L, with an average TSH level of 16.2 mIU/L. Also, antibodies against thyroid peroxidase (TPO) are found in up to 31% of patients with Down syndrome [1,8].

Screening for these values is recommended at birth, at six months, and annually after the age of one year [5,7], although screening is often not performed until one year after birth [5]. Even so, thyroid pathology continues to present earlier in patients with Down syndrome than in the general population, and it could be due to its high prevalence that pediatricians observe children’s glands more closely and diagnose their enlargement and dysfunction early [9]. For the detection of these hormones, it is recommended to measure TSH in dried blood drops and capillary sampling since these are techniques that can be easily performed and, thus, extend the screening [11].

The literature reviewed shows that many researchers express discrepancies regarding the initiation of treatment of subclinical hypothyroidism in these patients. The determination of specific reference values could be of great help in the decision to start replacement therapy or to maintain a wait-and-see attitude in these patients [3,6].

In various instances, potential hypothyroidism is observed, which may exhibit a transient nature [1,4,5,8]. Treatment is recommended solely if clinical symptoms persist over time or TSH elevation above 10 mIU/L is maintained [5]. The preferred therapeutic approach involves early administration of levothyroxine [5,7,8,12], especially in cases where TSH levels exceed 12 mIU/L [6]. Alternatively, disease stabilization can be achieved through methimazole if positive thyroid antibodies are detected [5].

Amr [1] reported that early thyroxine (T4) treatment in individuals with Down syndrome did not manifest benefits for motor or mental development; its positive impact was solely on growth. Moreover, he asserted that the presence of positive antithyroid antibodies does not justify immediate treatment, but requires strict thyroid monitoring, a strategy denoted as “watchful waiting” [1] and a stance supported by several authors.

There is a paucity of evidence regarding the potential benefits of folinic acid (FA) and T4, either in combination or administered independently, for patients [13]. What is recommended is the early referral of children for detection and treatment when deemed necessary, with the aim of mitigating morbidity and mortality [14].

Given the aforementioned findings, a definitive consensus regarding the initiation or abstention from replacement therapy for subclinical hypothyroidism among patients with Down syndrome remains elusive. The main objective of the present work was to establish reference values for TSH and FT4 hormones for people with DS, which are different from those of the general population, to determine when to treat existing hypothyroidism. It is imperative to establish the upper and lower reference limits (URL and LRL) for TSH and FT4 with their respective confidence intervals (CIs) to delineate the normal ranges in this population. This includes an exploration of whether age- and sex-specific reference intervals (RIs) are requisite. Such determinations would help to mitigate the iatrogenic effects evident in numerous cases.

## 2. Materials and Methods

### 2.1. Study Subjects

A retrospective study was conducted, encompassing 503 subjects with DS. The data collected were obtained from the Health Program for People with Down Syndrome in the Valencian Community [15], spanning the period from February 1993 to November 2021. Sample analyses were conducted uniformly since 2017, employing identical extraction and laboratory determination protocols. This study was approved by the Research Ethics Committee of the Catholic University of Valencia (approval code 2021–2022/186), and written informed consent was obtained from all participants. This work complied with the principles established in the Declaration of Helsinki.

Patients with DS of both sexes and of any age who attended the Health Program for specialized care were included in this study. To adhere as strictly as possible to the objectives of the analysis, patients with alterations in thyroid function, both clinical and analytical, were excluded. Applying these exclusion criteria, we configured a sample with a theoretically normal performance of glandular function.

Thus, the inclusion criteria used were the condition of DS and normal thyroid gland functionality; the exclusion criteria, on the one hand, included diagnosed thyroid pathology, with this being hyperthyroidism, or the presence of thyroid nodules or tumors of the gland. Likewise, it was considered an exclusion criterion if a patient was being treated with the following drugs: heparin, glucocorticoids, beta-blockers, or amiodarone-type antiarrhythmics (due to their possible interference with iodine metabolism).

In short, with the application of these inclusion and exclusion criteria, only those patients who had undergone clinical evaluation and who were reported as euthyroid in the clinical records of the Health Program for persons with Down syndrome, without distinction as to age or sex, formed part of the study.

### 2.2. Variables

This study incorporated socio-demographic variables, including age and sex, alongside analytical variables, namely Thyroid-Stimulating Hormone (TSH) and Free Thyroxine (FT4).

### 2.3. Laboratory Methods

TSH and FT4 determinations were performed using TSH3-Ultra ADVIA Centaur and FT4 ADVIA Centaur XP assays, both from Siemens Healthineers. TSH values are expressed as milli-International Units per liter (mIU/L), while FT4 values are expressed as nanograms per deciliter (ng/dL). The ADVIA Centaur manufacturer’s reference data for the general population are 0.25–5.00 mIU/L (TSH) and 0.9–1.7 ng/dL (FT4).

### 2.4. Data Analysis

Statistical analyses were carried out using R software (version 4.3.0). The normality of data distribution was assessed by using the Shapiro–Wilk test. Outliers were detected using the Reed criterion [16]. Due to non-normal distributions, data are presented as medians (lower and upper quartiles). Qualitative variables are expressed as absolute values and percentages (%). The comparison between the values was performed by using the Mann–Whitney test. The URL and LRL were established following the CLSI EP28-A3c [17] and the Spanish Society of Clinical Biochemistry and Molecular Pathology (SEQC) [18] guidelines using the 2.5th and 97.5th percentiles.

The medians, 2.5th and 97.5th percentiles, and 90% confidence interval (CI) of LRL and URL were estimated for TSH and FT4 using quantile regression models [19,20], considering the values of both hormones as independent, and including only the intercept term. To assess the influence of age and sex on URL and LRL, quantile regression models were performed for the 2.5th and 97.5th percentiles of the distribution, including both variables as dependent variables. The Wald test was employed to determine if URL and LRL remained constant across age and both sexes. Additionally, CI/RI ratios were calculated to compare the relative width of CIs and the accuracy of their estimation [21]. A two-sided *p*-value < 0.05 was considered statistically significant.

This study is reported following the Strengthening the Reporting of Observational Studies in Epidemiology (STROBE) guideline (available as Table S1 in Supplementary Material) [22].

## 3. Results

The sample analyzed included 503 people with Down syndrome. The mean age was 19.69 years (SD = 13.83). A total of 277 (55.1%) patients were male and 226 (44.9%) were female. Table 1 describes the sociodemographic and clinical characteristics of the sample.

The distributions of TSH and FT4, along with the URL and LRL of the IRs, their related 90% CIs, and the ratio of CI width to RI width, used to assess the relative uncertainty of the estimates are shown in Table 2.

**Figure 1:**
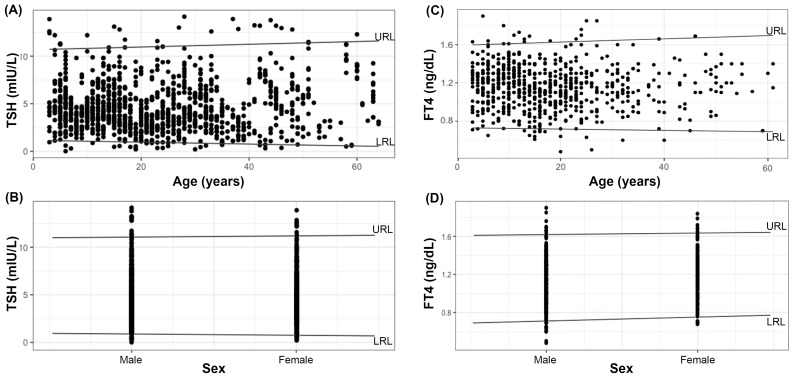
Trends of LRL and URL for TSH and FT4 according to age (**A**,**C**) and sex (**B**,**D**). Black dots represent the individual values of the hormones.

The values of the CI/RI ratios are less than 15% in all cases, suggesting an adequate sample size and that the data do not include values that could have a large influence on the regression fit and, therefore, on the reference limits. Figure 1 shows the LRL and URL trends for TSH and FT4 as a function of age and sex. Table 3 shows the results of the Wald test, which was found to be non-significant in all cases, suggesting that age and sex do not influence the distribution of quantiles.

**Figure 2:**
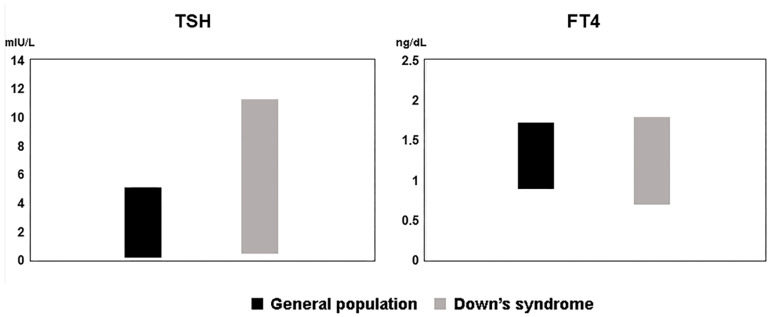
Comparison of reference values in patients with Down syndrome and in the general population.

Figure 2 shows the comparison of the reference values found with respect to the values established for the general population.

## 4. Discussion

The Health Program for People with Down Syndrome [15] is part of the activities for the health promotion and prevention of the pathologies prevalent in this group. The need to establish or develop a specific health program arises from the biological reality of DS itself. As an alteration of chromosome 21 (trisomy in most cases), the genetic anomaly unbalances the function of numerous genes, putting the full development and function of various organs and systems of the body at risk at any stage of life. The evaluation of these individuals includes analyses provided by users and requested by their pediatricians and family doctors. In most cases, particularly from the second visit onwards, the following are analyzed: hemogram, basic blood biochemistry, hepatorenal function, dyslipidemia, vitamins, iron metabolism, coeliac screening if applicable, and thyroid function.

The study of thyroid function is mandatory in these individuals due to the known association between Down syndrome (DS) and thyroid abnormalities [23]. This syndrome is linked with a high prevalence of subclinical hypothyroidism [24], most likely due to the extra chromosome 21 [12] or abnormal TSH generation due to other cardiac or intestinal diseases [25]. Furthermore, there is a very high possibility that hypothyroid symptomatology (especially if mild) may go unnoticed in the context of DS. Patients with DS commonly present, associated with their genetic condition, a constipated defecatory habit, a high body mass index, overweight or obesity, intolerance to cold, and marked xerosis (especially in the extremities), in addition to the cognitive deficit associated with the syndrome. All these symptoms, including bradypsychia, can be part of the clinical picture of hypothyroidism. Outside the neonatal period, the signs of hypothyroidism can be very faint in people with DS and can be confused with the clinical manifestations of the syndrome, leading to overlooked cases.

Many clinical practice guidelines for the management of hypothyroidism in adults consulted [26,27,28,29,30,31,32,33,34] indicate that DS is a risk factor for hypothyroidism, requiring special attention. Additionally, most of the time when a patient with Down syndrome is diagnosed with subclinical hypothyroidism, elevated antithyroid antibodies are found [1,8,35], suggesting autoimmunity associated with Hashimoto’s thyroiditis (HD) or Graves’ disease (GD) [5,7,9], which is another factor to consider when treating and diagnosing these patients [26]. In routine clinical practice, thyroid function is monitored by analyzing TSH and FT4 determinations. When FT4 is below range, the diagnosis is clear and the indication for replacement therapy is also evident. In most cases, TSH will be elevated (except in cases of hypothalamic insufficiency). The problem arises in cases where, with FT4 in the normal range, elevated TSH values (Subclinical Hypothyroidism) are found. Here, the algorithms of the guidelines are clear and, in most cases, indicate the initiation of treatment from TSH values that are double the maximum range (10 over 5). Similarly, the indications for requesting antithyroid antibodies to complete the diagnosis of Autoimmune Hypothyroidism (Hashimoto’s) are included. However, it is common in clinical practice to initiate TSH “suppression” treatment with values that exceed the upper range (although they do not reach 10), especially in the pediatric age group (infants and preschoolers). Indeed, the determination of antibodies is of great importance in the diagnosis of thyroid pathology, especially in the case of autoimmune diseases; however, in routine practice, fundamentally in primary care, access to these determinations is not always as easy as to those of TF4 and TSH values. In fact, therapeutic decisions in this area are commonly made with these measurements alone. Levothyroxine dosage adjustments are made exclusively with TSH. For this reason, despite the important limitation in the lack of data regarding antibodies, we believe that the results obtained continue to be relevant because they are adjusted to the context of routine diagnostic and therapeutic care, especially in primary care.

In the case of individuals with DS, it is very common to find TSH values in ranges above the values established as a reference for the general population, but FT4 figures are maintained in the normal range. On these occasions, it is decided to maintain expectant treatment, and it is observed that these TSH values always remain above the reference over the years, but without a progressive rise and sometimes even with downward fluctuations concerning previous analyses, and with the maintenance of FT4 always in the normal range. On the one hand, this could indicate a very slowly developing subclinical hypothyroidism, but, on the other hand, there is consideration that the normal TSH value in these individuals could be higher than that in individuals without DS.

The importance of the relationship between patients with Down syndrome and congenital thyroid dysfunction [36,37] justifies screening at birth, at six months [5,7], and from one year of age onwards annually in order to carry out an exhaustive control of this pathology [38], together with the antithyroid antibody count [1,2,4,8,35].

The main objective of the present work is to determine when to treat existing hypothyroidism in these patients [1,5], as it has been observed that the standard values used in the general population mainly diagnose subclinical hypothyroidism with a TSH measurement > 5 mlU/L [1,4,7]. In this regard, there is great controversy among different authors, such as Cebeci et al. [10] and Amr [1], on whether or not to treat hypothyroidism in these patients after finding very high TSH values without obvious symptomatology [1]. For this reason, reference values were established in this study, which we consider to be of great importance to maintain an attitude of watchful waiting for DS patients before starting replacement therapy unnecessarily. Although thyroid hormone levels in the general population have been reported to vary with age and sex [39,40,41], our results show that in the DS population, they remain constant despite being a heterogeneous population, so they can be considered unique for any age and sex.

Moreover, the normal values found for both parameters (TSH and FT4) in patients already diagnosed and being treated with levothyroxine will also serve for their control and adjustment since, in many cases, only TSH is analyzed, which, in these patients, could be “normally” elevated and yet maintain adequate blood FT4 levels, making it unnecessary to amend the doses of levothyroxine.

This situation frequently occurs in the primary care setting. Practitioners are sometimes forced to make therapeutic adjustment decisions, having only access to analytical determinations of FT4 and TSH (even sometimes only with TSH). In many cases, in this context, the decrease in TSH values (TSH suppression therapy) is used as the main target, especially in asymptomatic cases or in cases with subclinical hypothyroidism. This motivation drove the approach of this study: to achieve the establishment of standard values for hormone determinations that would help in the design of therapeutic strategy for people with Down syndrome, especially in the primary care setting.

The main limitation of this study was the absence of data on elevated antithyroid antibodies in order to rule out autoimmunity associated with Hashimoto’s thyroiditis or Graves’ disease. However, we tried to mitigate this as much as possible with the sample exclusion criteria. The determination of standard values for TSH and T4L, considered the key determinations in the control of thyroid pathology, conditioned the selection of patients in our study to form a sample with theoretically normal performance of glandular function. For this reason, as indicated above, we insisted on the exclusion criteria related to the presence of diagnosed thyroid pathology (hyperthyroidism, producing nodules, tumors, etc.) or receiving treatment with drugs that interfere with iodine metabolism (heparin, glucocorticoids, beta-adrenergic blockers, amiodarone, etc.). In contrast, the strengths of our study lie in the representativeness of the sample size and the use of Reed’s criterion [16] for the detection of outliers, as well as the robust statistical approach with the use of quantile regressions with the 2.5–97.5 fractional [17,18,19,20,21].

## 5. Conclusions

The determination of blood hormone values for monitoring thyroid function is especially important in the health assessment of individuals with DS, given the high prevalence of thyroid gland pathologies in this population. In routine clinical practice, especially in the primary care setting, it is common to have only FT4 and TSH values (sometimes only TSH).

The establishment of reference values for TSH and FT4 determinations in the blood of individuals with DS can be of great importance, both in diagnosis and in controlling the dose of replacement therapy in both clinical and, especially, subclinical hypothyroidism. After analyzing the data obtained in our sample, the identified interval is 0.88–11.25 mIU/L for TSH and 0.71–1.63 ng/dL for FT4, with the emphasis on the value of the upper limit of the TSH interval. These values, which can be considered unique for any age and sex, will serve to maintain an attitude of expectant vigilance over individuals with DS before initiating replacement therapy unnecessarily or for the control and adjustment of medication in diagnosed cases. This is particularly crucial when making these decisions in a clinical context with access to TSH values only.

## Figures and Tables

**Table 1:** Sociodemographic and clinical characteristics of the sample.

	Median (IQR) or *n* (%)	*p*-Value *
Gender		
Male	277 (55.1)	
Female	226 (44.9)	
TSH (mUI/L)	4.14 (2.72–6.08)	
Male TSH	4.08 (2.71–6.09)	0.939
Female TSH	4.20 (2.75–6.05)	
FT4 (ng/dL)	1.18 (1.00–1.31)	
Male FT4	1.13 (0.98–1.30)	0.484
Female FT4	1.20 (1.10–1.35)	

IQR: Interquartile Range; * Mann–Whitney test.

**Table 2:** TSH and FT4 distributions.

	Median (IQR)	95% RI	Lower Limit		Upper Limit	
			90% CI	CI/RI%	90% CI	CI/RI%
TSH (mUI/L)	4.15 (2.75–6.01)	0.88–11.25	0.73–1.03	2.9%	10.49–12.00	14.56%
FT4 (ng/dL)	1.16 (1.00–1.30)	0.71–1.63	0.67–0.75	8.7%	1.58–1.68	10.86%

**Table 3:** Results of LRL and URL comparison for TSH and FT4 as a function of age and sex.

Model	F-Value	*p*-Value *
TSH~Age (years)	0.5694	0.451
TSH~Sex	0.2982	0.585
FT4~Age (years)	0.7877	0.375
FT4~Sex	0.4903	0.484

* *p*-value between URL and LRL was determined from Wald tests and reflects no evidence for differences between them.

## Data Availability

Data are contained within the article.

## Supplementary Materials

The following supporting information can be downloaded at https://www.mdpi.com/article/10.3390/diagnostics13233499/s1. Table S1: Strobe Statement—checklist of items that should be included in reports of observational studies.

## References

[1] Amr N.H. (2018). Thyroid disorders in subjects with Down syndrome: An update. Acta Biomed..

[2] Campos C., Casado A. (2015). Oxidative stress, thyroid dysfunction & Down syndrome. Ind. J. Med. Res..

[3] Yahia S., El-Farahaty R.M., El-Hawary A.K., A El-Hussiny M., Abdel-Maseih H., El-Dahtory F., El-Gilany A.-H. (2012). Leptin, insulin and thyroid hormones in a cohort of Egyptian obese Down syndrome children: A comparative study. BMC Endocr. Disord..

[4] Prasher V. (1999). Down Syndrome and Thyroid Disorders: A Review. Down Syndr. Res. Pract..

[5] Whooten R., Schmitt J., Schwartz A. (2018). Endocrine manifestations of Down syndrome. Curr. Opin. Endocrinol. Diabetes Obes..

[6] Marchal J.P., Maurice-Stam H., Ikelaar N.A., Klouwer F.C.C., Verhorstert K.W.J., Witteveen M.E., Houtzager B.A., Grootenhuis M.A., van Trotsenburg A.S.P. (2014). Effects of early thyroxine treatment on development and growth at age 10.7 years: Follow-up of a randomized placebo-controlled trial in children with Down’s syndrome. J. Clin. Endocrinol. Metab..

[7] AlAaraj N., Soliman A.T., Itani M., Khalil A., De Sanctis V. (2019). Prevalence of thyroid dysfunctions in infants and children with Down Syndrome (DS) and the effect of thyroxine treatment on linear growth and weight gain in treated subjects versus DS subjects with normal thyroid function: A controlled study. Acta Biomed..

[8] King K., O’gorman C., Gallagher S. (2014). Thyroid dysfunction in children with Down syndrome: A literature review. Ir. J. Med. Sci..

[9] Aversa T., Crisafulli G., Zirilli G., De Luca F., Gallizzi R., Valenzise M. (2018). Epidemiological and clinical aspects of autoimmune thyroid diseases in children with Down’s syndrome. Ital. J. Pediatr..

[10] Ayşe N.C., Ayla G., Metin Y. (2013). Perfil de hipotiroidismo en el síndrome de Down. J. Clin. Res. Pediatr. Endocrinol..

[11] E Noble S., Leyland K., A Findlay C., E Clark C., Redfern J., Mackenzie J.M., A Girdwood R.W., Donaldson M.D.C. (2000). School based screening for hypothyroidism in Down’s syndrome by dried blood spot TSH measurement. Arch. Dis. Child..

[12] Zwaveling-Soonawala N., Witteveen M.E., Marchal J.P., Klouwer F.C.C., Ikelaar N.A., Smets A.M.J.B., van Rijn R.R., Endert E., Fliers E., van Trotsenburg A.S.P. (2017). Early thyroxine treatment in Down syndrome and thyroid function later in life. Eur. J. Endocrinol..

[13] Mircher C., Sacco S., Bouis C., Gallard J., Pichot A., Le Galloudec E., Cieuta C., Marey I., Greiner-Mahler O., Dorison N. (2020). Thyroid hormone and folinic acid in young children with Down syndrome: The phase 3 ACTHYF trial. Genet. Med..

[14] Yaqoob M., Manzoor J., Hyder S.N., Sadiq M. (2019). Congenital heart disease and thyroid dysfunction in down syndrome reported at children’s hospital, lahore, pakistan. Turk. J. Pediatr..

[15] Tomàs Aguirre F., Garrido López P., Martinez Borrel J.M., Vivat Moreno E., Rauet Corretger J.M., Cerdá Derlga-do-Fernandez R., Moldenhaeur Diaz F. (2020). Programa Español de Salud Para Personas con Síndrome de Down. DOWN ESPAÑA. https://www.sindromedown.net/wp-content/uploads/2021/10/PROGRAMA-SALUD_corr.pdf.

[16] Reed A.H., Henry R.J., Mason W.B. (1971). Influence of Statistical Method Used on the Resulting Estimate of Normal Range. Clin. Chem..

[17] CLSI (2010). Defining, Establishing, and Verifying Reference Intervals in the Clinical Laboratory.

[18] SEQC Sociedad Española de Bioquímica Clínica y Patología Molecular. http://www.seqc.es/.

[19] Yu K., Lu Z., Stander J. (2003). Quantile regression: Applications and current research areas. J. Roy. Stat. Soc..

[20] Boucai L., Hollowell J.G., Surks M.I., Amouzegar A., Bakhtiyari M., Mansournia M.A., Etemadi A., Mehran L., Tohidi M., Azizi F. (2011). An Approach for Development of Age-, Gender-, and Ethnicity-Specific Thyrotropin Reference Limits. Thyroid.

[21] Virtanen A., Kairisto V., Uusipaikka E. (1998). Regression-based reference limits: Determination of sufficient sample size. Clin. Chem..

[22] STROBE (2007). STROBE Checklist for Cohort Studies. https://www.strobe-statement.org/fileadmin/Strobe/uploads/checklists/STROBE_checklist_v4_cohort.pdf.

[23] Kennedy R.L., Jones T.H., Cuckle H.S. (1992). Down’s syndrome and the thyroid. Clin. Endocrinol..

[24] Purdy I.B., Singh N., Brown W.L., Vangala S., Devaskar U.P. (2014). Revisiting early hypothyroidism screening in infants with Down syndrome. J. Perinatol..

[25] van Trotsenburg A.P., Heymans H.S., Tijssen J.G., de Vijlder J.J., Vulsma T. (2006). Comorbidity, Hospitalization, and Medication Use and Their Influence on Mental and Motor Development of Young Infants with Down Syndrome. Pediatrics.

[26] Garber J.R., Cobin R.H., Gharib H., Hennessey J.V., Klein I., Mechanick J.I., Pessah-Pollack R., Singer P.A., Woeber K.A., American Association of Clinical Endocrinologists and American Thyroid Association Taskforce on Hypothyroidism in Adults (2012). Clinical Practice Guidelines for Hypothyroidism in Adults: Cosponsored by the American Association of Clinical Endocrinologists and the American Thyroid Association. Endocr. Pract..

[27] Ross D.S., Burch H.B., Cooper D.S., Greenlee M.C., Laurberg P., Maia A.L., Rivkees S.A., Samuels M., Sosa J.A., Stan M.N. (2016). 2016 American Thyroid Association Guidelines for Diagnosis and Management of Hyperthyroidism and Other Causes of Thyrotoxicosis. Thyroid.

[28] Guía Clínica de Hipotiroidismo Subclínico—Fisterra. https://www.fisterra.com/guias-clinicas/hipotiroidismo-subclinico/.

[29] Noorily S.H. (2008). Hipotiroidismo. Toma de Decisiones en Anestesiología.

[30] Corrales Hernández J.J., Alonso Pedrol N., Cantón Blanco A., Galofré Ferrater J.C., Pérez Pérez A., Lajo Morales T., Perez Corral B., Henzi F.T. (2007). Guía clínica del diagnóstico y tratamiento de la disfunción tiroidea subclínica. Endocrinol. Nutr..

[31] Guía Clínica Manejo de las Alteraciones Funcionales y Morfológicas del Tiroides. https://www.comcordoba.com/wp-content/uploads/2019/06/GU%C3%8DA-CL%C3%8DNICA-patolog%C3%ADa-del-Tiroides-A.S.-Norte-C%C3%B3rdoba.pdf.

[32] Rodrigues A.L., Carvalho A., Pereira Duarte C., César R., Anselmo J. (2014). Hipotiroidismo congénito. Rev. Port. Endocrinol. Diabetes Metab..

[33] Spanish Society of Endocrinology and Nutrition Thyroid. https://www.seen.es/portal/presentacion-areas-conocimiento-endocrinologia.

[34] (2018). Spanish Society of Pediatric Endocrinology—Thyroid. https://www.seep.es/index.php/recursos-ep/tiroides.

[35] Murdoch J.C., Ratcliffe W.A., Mclarty D.G., Rodger J.C., Ratcliffe J.G. (1977). Thyroid Function in Adults with Down’s Syndrome. J. Clin. Endocrinol. Metab..

[36] Lavigne J., Sharr C., Elsharkawi I., Ozonoff A., Baumer N., Brasington C., Cannon S., Crissman B., Davidson E., Florez J.C. (2017). Thyroid dysfunction in patients with Down syndrome: Results from a multi-institutional registry study. Am. J. Med. Genet. Part A.

[37] Ergaz-Shaltiel Z., Engel O., Erlichman I., Naveh Y., Schimmel M.S., Tenenbaum A. (2017). Neonatal characteristics and perinatal complications in neonates with Down syndrome. Am. J. Med. Genet. Part A.

[38] Bunt C.W., Bunt S.K. (2014). Role of the family physician in the care of children with Down syndrome. Am. Fam. Physician.

[39] Płaczkowska S., Terpińska M., Piwowar A. (2022). Establishing laboratory-specific reference intervals for TSH and fT4 by use of the indirect Hoffman method. PLoS ONE.

[40] Adeli K., Higgins V., Trajcevski K., Habeeb N.W.-A. (2017). The Canadian laboratory initiative on pediatric reference intervals: A CALIPER white paper. Crit. Rev. Clin. Lab. Sci..

[41] Park S.Y., Kim H.I., Oh H.-K., Kim T.H., Jang H.W., Chung J.H., Shin M.-H., Kim S.W. (2018). Age- and gender-specific reference intervals of TSH and free T4 in an iodine-replete area: Data from Korean National Health and Nutrition Examination Survey IV (2013–2015). PLoS ONE.

