# Supplementary material for: Is There an Adequate Therapeutic Approach to Thyroid Pathology in Patients with Down Syndrome?
Source: Diagnostics (Basel). 2023 Nov 21;13(23):3499. doi: 10.3390/diagnostics13233499 (PMC10706773; doi:10.3390/diagnostics13233499)
Supplement: Supplementary file 1 [file diagnostics-13-03499-s001.zip › diagnostics-2715904-supplementary.pdf]

Table S1. STROBE Statement—checklist of items that should be included in reports of observational studies

	Item No.	Recommendation	Section and paragraph numbers
**Title and abstract**	1	(*a*) Indicate the study's design with a commonly used term in the title or the abstract	title
		(*b*) Provide in the abstract an informative and balanced summary of what was done and what was found	abstract
**Introduction**			
Background/rationale	2	Explain the scientific background and rationale for the investigation being reported	Introduction, paragraph 1-10
Objectives	3	State specific objectives, including any prespecified hypotheses	Introduction, paragraph 11-12
**Methods**			
Study design	4	Present key elements of study design early in the paper	Study design and participants, paragraph 1-2
Setting	5	Describe the setting, locations, and relevant dates, including periods of recruitment, exposure, follow-up, and data collection	Study design and participants, paragraph 1-2
Participants	6	(*a*) Give the eligibility criteria, and the sources and methods of selection of participants. Describe methods of follow-up	Study design and participants, paragraph 2
		(*b*) For matched studies, give matching criteria and number of exposed and unexposed	NA
Variables	7	Clearly define all outcomes, exposures, predictors, potential confounders, and effect modifiers. Give diagnostic criteria, if applicable	Laboratory Methods
Data sources/ measurement	8*	For each variable of interest, give sources of data and details of methods of assessment (measurement). Describe comparability of assessment methods if there is more than one group	Laboratory Methods
Bias	9	Describe any efforts to address potential sources of bias	Study design and participants, paragraph 2
Study size	10	Explain how the study size was arrived at	Study design and participants, paragraph 1

Continued on next page

Quantitative variables	11	Explain how quantitative variables were handled in the analyses. If applicable, describe which groupings were chosen and why	Data analysis
Statistical methods	12	(*a*) Describe all statistical methods, including those used to control for confounding	Data analysis
		(*b*) Describe any methods used to examine subgroups and interactions	NA
		(*c*) Explain how missing data were addressed	NA
		(*d*) If applicable, explain how loss to follow-up was addressed	NA
		(*e*) Describe any sensitivity analyses	NA
**Results**			
Participants	13*	(a) Report numbers of individuals at each stage of study—eg numbers potentially eligible, examined for eligibility, confirmed eligible, included in the study, completing follow-up, and analysed	Study design and participants, paragraph 1
		(b) Give reasons for non-participation at each stage	Study design and participants, paragraph 1
		(c) Consider use of a flow diagram	NA
Descriptive data	14*	(a) Give characteristics of study participants (eg demographic, clinical, social) and information on exposures and potential confounders	Results, paragraph 1
		(b) Indicate number of participants with missing data for each variable of interest	NA
		(c) *Cohort study*—Summarise follow-up time (eg, average and total amount)	Results, paragraph 1
Outcome data	15*	*Cohort study*—Report numbers of outcome events or summary measures over time	Results, paragraph 1
Main results	16	(*a*) Give unadjusted estimates and, if applicable, confounder-adjusted estimates and their precision (eg, 95% confidence interval). Make clear which confounders were adjusted for and why they were included	Results, paragraph 2-4<br>Figure 1
		(*b*) Report category boundaries when continuous variables were categorized	Table 1, Table 2
		(*c*) If relevant, consider translating estimates of relative risk into absolute risk for a meaningful time period	NA

Continued on next page

Other analyses	17	Report other analyses done—eg analyses of subgroups and interactions, and sensitivity analyses	Results, Table 3, Figure 2
**Discussion**			
Key results	18	Summarise key results with reference to study objectives	Discussion, paragraph 1
Limitations	19	Discuss limitations of the study, taking into account sources of potential bias or imprecision. Discuss both direction and magnitude of any potential bias	Discussion, paragraph 1-6
Interpretation	20	Give a cautious overall interpretation of results considering objectives, limitations, multiplicity of analyses, results from similar studies, and other relevant evidence	Discussion, paragraph 7
Generalisability	21	Discuss the generalisability (external validity) of the study results	Discussion, paragraph 8
**Other information**			
Funding	22	Give the source of funding and the role of the funders for the present study and, if applicable, for the original study on which the present article is based	NA
